# Effects of a Dietary Blend of Essential Oils, Capsaicin, and Yeast Metabolites on Performance, Physiological, Metabolism, and Immune Response of Heat-Stressed Pigs

**DOI:** 10.3390/vetsci12100976

**Published:** 2025-10-11

**Authors:** Lorena Duarte Campos, Danilo Alves Marçal, Ismael França, Cleslei Alisson Silva, Alini Mari Veira, Amanda Faria Oliveira, Alícia Zem Fraga, Rafael C. de Araujo, Alex Sandro Campos Maia, Luciano Hauschild

**Affiliations:** 1 Department of Animal Science, School of Agricultural and Veterinary Sciences (FCAV), São Paulo State University (UNESP), Via de Acesso Prof. Paulo Donato Castellane, s/n, Jaboticabal 14884-900, Brazil; 2 Graduate Program in Animal Production, Universidade Brasil, Fernandópolis 15600-000, Brazil; 3 Department of Animal Science, Universidade Federal Rural do Rio de Janeiro, Seropédica 23890-000, Brazil; 4 GRASP Industry and Commerce Ltd., Curitiba 81260-000, Brazil

**Keywords:** high ambient temperature, nutritional strategy, plant extracts, swine nutrition

## Abstract

**Simple Summary:**

High environmental temperatures can negatively impact pig health, growth, and metabolism. Heat-stressed pigs often reduce their feed intake and experience impaired nutrient absorption, increased fat deposition, and inflammation, all of which compromise growth performance. Natural feed additives, such as compounds derived from essential oils, yeast metabolites, and capsaicin (a compound from chili peppers), have shown potential to support gut health, modulate immune responses, and improve animal performance under stressful conditions. This study investigated the effects of a dietary blend containing essential oils, capsaicin, and yeast metabolites on pigs raised under chronic heat stress. Pigs were monitored for growth, body composition, blood markers, and metabolism over 56 days at a constant high temperature (35 °C). While the additive did not improve overall performance, pigs fed the blend tended to have reduced fat deposition and showed changes in some biochemical indicators related to metabolism. These findings suggest that although this specific blend did not fully mitigate the effects of heat stress, it may influence fat metabolism and deserves further investigation. Understanding how natural additives affect pigs under heat stress can help develop nutritional strategies to support animal health and productivity in increasingly warm climates.

**Abstract:**

This study investigated the effects of a dietary additive composed of compounds derived from essential oils (carvacrol, eugenol, cinnamaldehyde), capsaicin, and yeast metabolites on the performance, body composition, metabolism, and immune status of pigs under chronic heat stress (HS). A total of 24 crossbred gilts (50 ± 3.98 kg) were assigned to one of two diets: a control diet (CON) or the same diet supplemented with the additive blend (2.5 g/kg; BLEND). Animals were housed collectively, with individual feed intake recorded using automatic precision feeders over a 56-day period at a constant ambient temperature of 35 °C. Heat stress increased rectal temperature initially (*p* < 0.01), which gradually declined over time. No significant differences were found in overall performance or tissue deposition between treatments (*p* > 0.05), though pigs fed the BLEND diet tended to have an 18% lower fat deposition compared with the CON group (148.3 vs. 121.3 g/d, *p* = 0.094). The additive had no effect on inflammatory or most biochemical parameters, except for increased creatinine compared with the CON group (1.76 vs. 1.63 mg/dL; *p* = 0.032) and a tendency for elevated LDH (1064.87 vs. 939.17 U/L; *p* = 0.075). In conclusion, chronic HS impaired metabolic and immune parameters and altered body composition. The dietary blend did not enhance performance but showed a tendency to reduce lipid deposition under thermal stress conditions. Further studies are needed to elucidate the individual and combined actions of this feed additive in mitigating the impacts of HS on pigs.

## 1. Introduction

High ambient temperatures negatively affect animal health and growth performance. In response to HS, pigs reduce their feed intake to decrease metabolic heat production [1] and increase their peripheral blood flow to increase heat dissipation. These mechanisms lead to nutrient and oxygen limitation in the gastrointestinal tract, resulting in damage to intestinal integrity [2] and alterations in nutrient absorption [2,3]. In addition, impaired intestinal permeability allows the infiltration of pathogens and translocation of endotoxins into the blood, stimulating an inflammatory response [4]. Activation of the immune system has a high energy requirement and induces metabolic and physiological changes that affect growth performance [5].

In addition, HS increases fat deposition in pigs. This mechanism may be related to an increase in circulating insulin in HS conditions, an antilipolytic hormone, leading to inhibition of lipolysis [6,7] despite an insulin-resistant state [8]. Indeed, high ambient temperatures increase backfat lipid content and lipid metabolic activity [9] and attenuate lipid mobilization in pigs [10]. Moreover, HS limits body protein synthesis, resulting in greater lipid deposition when pigs under HS are fed ad libitum [11].

Nutritional strategies with natural feed additives may alleviate the negative effects of HS. Essential oils have been studied as feed additive alternatives due to their antioxidant, antimicrobial, and gut health-promoting properties [12,13]. Pigs raised under a high ambient temperature fed diet with 0.01% inclusion of essential oil (*Cinnamomum cassia*) had a greater average daily gain and reduced serum cortisol concentrations compared with pigs in thermoneutral condition [14]. Yeast metabolites may also prevent pathogen growth, modulate the immune system, and promote intestinal health [15,16]. Some studies have shown that the addition 0.2 to 0.3% of yeast supply improved performance and carcass weight in heat-stressed pigs [17]. Another biologically active compound of interest for its antioxidant, anti-inflammatory, digestive, and immunostimulant properties is capsaicin (*Capsicum oleoresin*) [18]. This plant extract played a role in thermoregulatory responses, including cutaneous vasodilation and panting [19]. Capsaicin is an alkaloid found in several species of hot peppers and can be used as a palatability and flavoring agent. Moraes et al. [20] reported that the inclusion of 1.4 g/kg of capsaicin improved feed intake and IgG colostrum concentration in sows, improved litter weight gain, and reduced piglet diarrhea. When fed to weaned piglets, capsaicin reduced intestinal expression of inflammatory genes due to decreased immune response [21] and stimulated antioxidant activity [22].

Limited information is available on the combination of these dietary compounds during HS in pigs, and due to the beneficial effects of these natural substances, we hypothesized that their feed inclusion could attenuate the deleterious effects of HS on physiology, metabolism, and performance of growing and finishing pigs. Therefore, the aim of this study was to evaluate the effect of a dietary inclusion of a blend composed of compounds derived from essential oils (carvacrol, eugenol, and cinnamaldehyde), pepper extract rich in capsaicin, and yeast metabolites from sugar cane on the performance, body composition, metabolism, and immune status of growing and finishing pigs under chronic HS.

## 2. Materials and Methods

All procedures followed the Brazilian National Council of the Control of Animal Experimentation (CONCEA) and were approved by the São Paulo State University Institutional Animal Care and Use Committee, SP, Brazil (protocol code 4449/22). The experiment was carried out at the Swine Research Facilities at São Paulo State University (UNESP), School of Agricultural and Veterinary Sciences, Jaboticabal, SP, Brazil.

### 2.1. Animals, Housing, and Experimental Design

Twenty-four gilts [(50 ± 3.98 kg of initial BW, Camborough dams × AGPIC 337 sires (Agroceres PIC, Rio Claro, Brazil)] were collectively housed in a 23 m^2^ suspended pen (4.40 × 5.20 m) with plastic floors, equipped with six nipple drinkers and two automatic and intelligent precision feeders (AIPF; Exafan, San Mateo de Gállego, Spain). Each animal received an electronic ear tag for individual identification, which, when recognized by any AIPF, delivered feed in response to each animal’s request according to the assigned experimental diet. In addition to providing the diet established individually, the AIPF recorded the individual consumption of each animal. The functioning of these feed stations was previously described by Pomar et al. [23].

Pigs were blocked by initial BW, and each BW block was randomly assigned to one of two dietary treatments: the control diet (CON; *n* = 12) or diet with additive inclusion (BLEND; *n* = 12). Both diets were based on corn and soybean meal, supplemented with crystalline amino acids, minerals, and vitamins, formulated to meet the nutritional requirements of growing and finishing pigs according to the National Research Council [24] (Table 1).

The diets were prepared at the feed mill of the São Paulo State University (UNESP), School of Agricultural and Veterinary Sciences, and supplied in pelleted form. The feed additive (Amenus, GRASP Ind. & Com. LTDA, Curitiba, Brazil) was incorporated directly into the feed mixture prior to pelleting. The BLEND diet consisted of including a dosage of 2.50 g/kg of a feed additive composed of a blend of compounds derived from essential oils (pure forms of carvacrol, eugenol, and cinnamaldehyde), pepper extract rich in capsaicin, and yeast metabolites (*Sacchamoryces cerevisiae*) obtained from sugar cane fermentation. The feed additive consisted of 925 g/kg of yeast metabolites and 75 g/kg of the blend of plant secondary compounds that were previously microencapsulated. Major secondary compounds in the final feed additive formulation were 0.30% of eugenol and 0.23% of pepper extract. The final dosage of 2.50 g/kg of feed was selected based on the manufacturer’s recommendation for use under heat stress conditions and extrapolated from previous studies in cattle, adjusted for metabolic weight and species differences to ensure an effective and safe level for pigs.

The experimental period lasted 56 days, which consisted of two phases: phase 1 (0–28 days) and phase 2 (29–56 days). The ambient temperature was kept constant at 35 °C during the experimental period through an automated climate system composed of electric heaters and air conditioning. Room temperature was monitored using a data logger (HT-70, Instrutherm, São Paulo, Brazil).

Pigs were housed in the experimental room one week before the start of the trial for adaptation. This period allowed the animals to reach an average BW of 50 kg and adapt to the experimental facilities. During the adaptation period, ambient temperature was kept at 22 °C and the animals were fed a common diet. On day 0, ambient temperature was gradually increased until it reached 35 °C. Feed and water were provided ad libitum throughout the trial, including the adaptation and experimental periods.

### 2.2. Data Collection

#### 2.2.1. Temperature–Humidity Index

The temperature–humidity index (*THI*) was calculated and classified according to [25], using the following equation:

*THI* = (9/5 × *AT* + 32) − [11/20 − (11/20 × *RH*)] × (*AT* − 26), (1)

where *AT* is the average daily temperature, expressed in °C, and *RH* is the average daily relative humidity, expressed as a decimal fraction. Based on THI values, environmental conditions were classified as follows: *THI* 73–77, mild heat stress; *THI* 78–82, moderate heat stress; and *THI* ≥ 83, severe heat stress.

#### 2.2.2. Rectal Temperature Data Collection

From day 0 to 14, the rectal temperature (RT) of each animal was measured daily at 6:00 a.m. After this period, it was measured once a week until the end of the experimental period (days 21, 28, 35, 42, 49, and 56). RT was measured using digital clinical thermometer (TH150, G-Tech, Accumed-Glicomed, São Paulo, Brazil).

#### 2.2.3. Growth Performance and Body Composition Measurements

The animals were weighed individually at the beginning and end of each phase (days 0, 28, and 56) after overnight fasting to determine the average daily gain (ADG, kg/day). Average daily feed intake (ADFI, kg/day) and gain–feed ratio (G:F, kg/kg) were calculated by phase during the experimental period and by the overall experimental period (0–56 days). Feed intake data was obtained using the AIPFs.

To determine total-body lean and lipid mass, the body composition of the pigs was measured at days 0, 28, and 56 using dual-energy X-ray absorptiometry equipment (DXA; Lunar Prodigy Advance, GE Healthcare, Chicago, IL, USA). After overnight fasting (8 h), pigs were anesthetized and sedated by intramuscular injection of acepromazine (0.1 mg/kg), xylazine (1.5 mg/kg), and ketamine (15 mg/kg) and scanned in prone position (from head to tail). Body protein and lipid mass obtained with DXA were converted into their respective chemical equivalents using the equations reported by Pomar and Rivest [26]. Protein or lipid deposition was divided by the ADG to obtain protein or lipid in relation to weight gain (%).

#### 2.2.4. Blood Sampling Analysis

On days 0, 7, and 28, blood samples were collected via jugular venipuncture in serum tubes or K2EDTA tubes (BD Vacutainer, São Paulo, Brazil) from fasted pigs (6 h). These specific sampling days were selected to represent different physiological phases of heat stress: day 7 corresponds to the acute phase, characterized by marked metabolic responses, whereas day 28 represents the chronic phase, when pigs had partially adapted to heat stress conditions [27]. Serum and plasma were obtained after centrifugation at 1000× *g* for 10 min at 4 °C (NT 835, Novatecnica, Piracicaba, Brazil), separated into 1.5 mL microtubes, and stored at −80 °C until analyses. Plasma and serum samples were obtained to measure inflammatory response markers (acute-phase proteins) and for biochemical analysis. Serum acute-phase protein (APP; immunoglobulin A (IgA), immunoglobulin G (IgG), albumin, haptoglobin, and α-1 acid glycoprotein) concentrations were measured using a gel electrophoresis technique and corrected by total protein concentration. Serum and plasma metabolite (total protein, urea, creatinine, glucose, lactate dehydrogenase (LDH), lactate, and triglycerides) concentrations were evaluated using a biochemistry and turbidimetry semiautomatic spectrophotometer (Labmax Plenno, Labtest, Lagoa Santa, Brazil).

#### 2.2.5. Statistical Analysis

Data were analyzed as a randomized complete block design using PROC GLIMMIX in SAS 9.4 [28]. For performance and body composition parameters, dietary treatment was considered as fixed effect, and the BW block was considered as a random effect. For rectal temperature and blood parameters data were analyzed as repeated measurements over time, with an autoregressive [AR(1)] covariance structure to account for temporal dependencies. The dietary treatment and day of measurement, as well as their interactions, were considered fixed effects, while the BW block was considered as a random effect. The normality of the residuals was analyzed by the Shapiro–Wilk test using the UNIVARIATE procedure, and outliers were considered when the residuals were greater than 3 or less than −3. The animal was considered the experimental unit. *p*-values of *p* ≤ 0.05 and 0.05 < *p* ≤ 0.10 were considered significant differences and tendencies, respectively.

## 3. Results

During the experimental period, two pigs from the CON diet treatment were removed due to health problems not related to the experimental procedures: one with a foot infection and the other with an open hernia. The data of these animals were excluded from the database. The average ambient temperature remained reasonably constant (35 ± 3.55 °C), and the average air relative humidity was 59.1 ± 13.7% during the trial. The THI varied between 80 and 98, which, according to the established classification, indicates that the animals were exposed to severe heat stress throughout the experimental period.

### 3.1. Rectal Temperature

**Figure 1:**
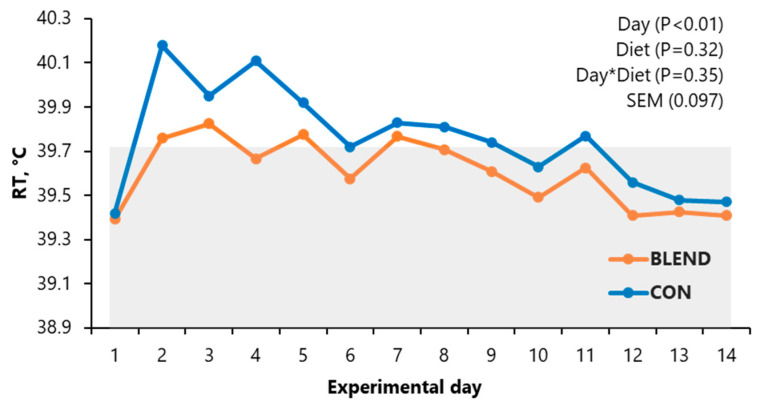
Average daily rectal temperature obtained during the first 14 days of phase 1 of growing–finishing gilts fed diets with the addition of an additive composed of a blend of compounds derived from essential oils, pepper extract rich in capsaicin, and yeast metabolites or not under constant heat stress. Means of RT outside the gray rectangle differ significantly according to the day of measurement (*p* < 0.01), and means of RT inside the gray rectangle indicate a non-significant difference (*p* > 0.05) on the day of measurement. RT: rectal temperature (°C); SEM: standard error of mean.

The exposure of pigs to heat stress conditions resulted in an increased RT on day 1 (*p* < 0.01; Figure 1), followed by a gradual reduction over time. From day 10 onwards, there were no differences in RT according to the day of measurement (*p* > 0.05). When rectal temperature data were evaluated on a weekly basis, pigs exhibited the highest RT values during the first week of heat stress exposure, with a clear peak on day 1 and a progressive decline thereafter. By weeks 2 and 3, RT values had stabilized and no longer differed significantly among treatments or over time until the end of experimental period (D56). There was no effect of dietary treatment on RT (*p* > 0.05) nor an interaction (*p* > 0.05) between dietary treatment and the day of measurement on RT.

### 3.2. Growth Performance and Body Composition

**Figure 2:**
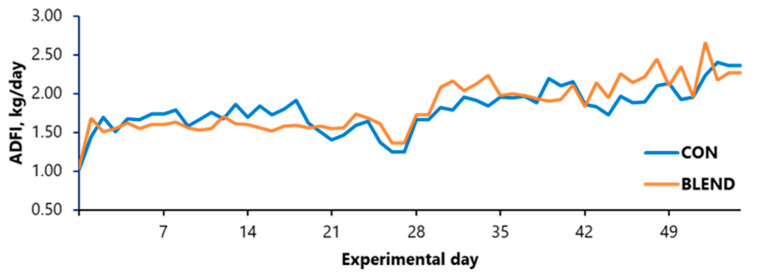
Overall average daily feed intake of growing–finishing gilts fed diets with the addition of an additive composed of a blend of compounds derived from essential oils, capsaicin-rich pepper extract, and yeast metabolites or not under constant heat stress. ADFI: average daily feed intake.

Effects of the diets on performance and body composition are shown in Table 2. In phase 1, pigs in the CON group tended to have a higher ADG than pigs fed the BLEND diet (0.70 vs. 0.65; *p* = 0.097), whereas ADFI, G:F, and tissue deposition were not affected by the dietary treatments. In phase 2, no significant differences were observed in performance, body composition, or tissue deposition between the CON and BLEND groups (*p* > 0.05), except for a tendency in the final body lipid. Pigs fed the BLEND diet tended to have lower body lipid than pigs fed the CON diet (14.61 vs. 16.35 kg; *p* = 0.10). During the overall period, pigs fed the BLEND diet tended to have lower lipid deposition compared with the CON group (121.3 vs. 148.3 g/d; *p* = 0.094). No significant effects were observed on ADG, ADFI (Figure 2), G:F, or protein deposition among dietary treatments (*p* > 0.05).

### 3.3. Blood Parameters

The results of serum and plasma metabolites concentrations are presented in Table 3. There was no interaction between diet and day of measurement for biochemical blood metabolites (*p* > 0.05). The heat stress condition until day 7 resulted in decreased concentrations of total protein, urea, creatinine, and LDH (*p* < 0.001), while glucose concentration increased (*p* < 0.001) on this day. Lactate concentration did not differ at the end of the first experimental week but was lower at day 28 (*p* < 0.001). Triglyceride concentration decreased on both days 7 and 28 compared with day 0 (*p* < 0.001). At day 28, total protein, urea, creatinine, and LDH concentrations increased compared with day 7 (*p* < 0.001), but the average concentration of these blood metabolites was still lower than at day 0, while glucose concentration at day 28 decreased when compared with day 7 (*p* < 0.001). Compared with pigs in the group CON, the BLEND group presented higher creatinine concentration, and LDH concentration tended to be higher (*p* = 0.032 and *p* = 0.075, respectively). The feed additive inclusion had no effect on the other serum and plasma metabolites measured (*p* > 0.05).

There was no interaction between diet and day of measurement for APP, neither was an effect of feed additive observed (Table 4; *p* > 0.05). When compared with day 0, serum IgA, IgG, albumin, and haptoglobin concentrations decreased after 7 days of heat stress (*p* < 0.001), except for α-1 acid glycoprotein concentration, which did not differ during this period. At day 28, the APP concentrations increased when compared with day 7 (*p* < 0.001).

## 4. Discussion

As the environmental heat load increases, thermoregulatory responses are triggered, resulting in productive and economic losses in the swine industry. One notable impact of HS is the increased lipid deposition, which compromises carcass composition at slaughter. Although advancements have been made in technologies such as ventilation, cooling systems, and nutritional strategies, HS remains one of the major challenges in swine production due to the intensification in climate change [27]. In this scenario, nutritional strategies represent an opportunity to ensure the long-term sustainability of animal production.

In this study, we hypothesized that adding a blend of compounds derived from essential oils (carvacrol, eugenol, and cinnamaldehyde), capsaicin-rich pepper extract, and yeast metabolites from sugar cane in the diet attenuate the negative effects of HS, by improving gut health and inflammatory responses, ultimately improving performance and body composition of pigs. Our main interesting findings revealed that pigs fed the dietary blend addition tended to have lower lipid deposition compared with those in the control, suggesting a potential attenuation of HS-induced changes in body composition.

The thermoneutral zone (TNZ) for growing–finishing pigs is 18–25 °C, which is the range of ambient temperature at which an animal can maintain its core temperature [29]. Within this zone, the animals’ heat production and dissipation mechanisms operate at basal levels, allowing them to maintain homeostasis without the need for adaptive thermogenesis (increased metabolic heat production) or evaporative cooling (e.g., panting) [30]. In our study, the ambient temperature was 35 ± 3.55 °C; the average relative humidity was 59.1 ± 13.7%. The average THI ranged from 80 to 95. Ref. [31] reported that a THI below 75 was considered normal for pigs and above 83 indicate severe heat stress. Thus, those parameters indicate that the animals in the current study were indeed exposed to HS conditions.

Above TNZ (i.e., heat stress), the animal will attempt to maintain its core temperature by decreasing heat production and increasing heat loss. The inability and/or adaptive response to maintain euthermia is translated into the behavior of RT (an indirect physiological indicator of core temperature). In the current study, the first day of the thermal challenge resulted in an increased RT followed by a gradual long-term reduction showing thermal acclimatation of the pigs exposed to the HS, as previously described [31,32,33]. The subsequent stabilization in RT does not indicate an absence of heat stress, but rather the activation of thermoregulatory and acclimatization mechanisms, such as increased peripheral blood flow, reduced feed intake, and hormonal adjustments, which allow pigs to partially maintain homeostasis despite the thermal challenge.

During HS, reduction in feed intake is a highly conserved response and represents an attempt to minimize metabolic heat production resulting in performance losses [34]. Blend inclusion did not improve performance and body composition of pigs under HS during the growing–finishing phase, except for a tendency in lower fat deposition on the overall period. Some studies have shown that the addition of natural ingredients (plant extracts or mixtures of plant extracts) have a positive impact on the physiology and gut health that results in an improvement on the growth performance [17,20,35]. However, there are reports that did not observe any effects on pig growth performance when plant extracts were added to the diets [36,37]. In the present study, the blend inclusion tended to induce a lower ADG in phase 1. The reasons for this result are not entirely clear. Even with a tendency of a lower ADG, animals fed the BLEND diet maintained similar protein deposition to the CON group throughout the study. At the end of the experimental period, both groups had a similar final BW, but the BLEND group presented lower lipid deposition. It is important to consider the magnitude of the heat-stress (HS) challenge, whether acute, chronic, or cyclic, when evaluating the animal’s response, as well as any effects from dietary or additive blends used. As an example, constant HS has a greater magnitude of negative consequences than cyclic HS [38]. In the current study, the duration (56 days) and the HS condition (35 °C) may have extrapolated the capacity of the blend to improve growth performance. This could explain the lack of blend effect, apart from the dosage and blend composition. Indeed, the effect of natural ingredients on animal performance varies widely, depending on dosage, plant species used, period of use, and animal housing conditions.

In terms of body composition, HS can reduce the digestibility of nutrients, reducing the nutrients available for protein deposition and increasing catabolism [39,40]. Baumgard and Rhoads [7] observed that HS elevates both basal and stimulated insulin levels, an antilipolytic hormone, which, despite contributing to an insulin-resistant state, inhibits adipose tissue mobilization and limits lipid release. Similarly, Sanz Fernandez et al. [41] observed that the upregulation of insulin secretion during HS increased insulin sensitivity in heat-stressed pigs when compared with pair-fed thermoneutral pigs. This mechanism is a response to spare glucose and supports the increased demand necessary to maintain glucose homeostasis [27]. Thus, with the impaired ability to mobilize lipids and the increased capacity for lipogenesis, this results in greater lipid deposition in pigs [10]. Although not evaluated in the current study, the blend may have influenced some pathway that impacted hormonal metabolism (insulin) with an influence on lipid metabolism. However, this still needs to be investigated. The lower lipid deposition with the same energy intake (same feed intake) compared with the CON group allowed more energy availability to be used for protein deposition. However, the protein deposition was similar between treatments. This may be related to the fact that while the deleterious effects of HS remain (e.g., activation of the immune response), the animal is not able to increase protein deposition. In fact, according to our results, the blend was not able to attenuate the impact of HS on immune activation. Nevertheless, the pigs fed the blend showed a tendency to lower body fat, which can be beneficial for markets that reward based on this.

HS exposure affected all plasma and serum metabolites, as well as APP. Our results are consistent with the altered post-absorptive metabolism characteristic of heat-stressed animals. On D7, the HS condition increased serum glucose concentration, and this response appears to be related to increased loads of glucose transporters SGLT1 and GLUT2 and higher glycogenolysis-related hepatic glucose process in pigs under HS [42,43]. In addition, the increased reduction in fatty acid oxidation under chronic HS becomes increasingly dependent on glucose to meet animal’s energy requirements [44]. In contrast, at high ambient temperature, lower glucose levels can also be found [6,37,43] due to the reduced feed intake. In our results, on D28, HS blood glucose levels are consistent with these findings, possibly explained by the prolonged phase of reduced feed intake and consequent decreased glucose absorption in the small intestine. The reduced serum concentration of urea, which is an indicator of tissue protein degradation, can also be related to the reduced feed intake induced by the HS (i. e. reduced protein intake) [45]. Changes in creatinine levels may also indicate altered protein catabolism [6]. Although higher creatinine levels may indicate protein breakdown, creatinine production is proportional to lean body mass [46], which may have reflected in higher creatinine concentrations on D28. The elevated creatinine levels observed in pigs fed the BLEND compared with the CON group may be linked to increased energy demands for lipid mobilization, and to the conversion of creatine to creatinine. Creatine is essential for energy metabolism through conversion to creatine phosphate by creatine kinase in the organs and it is also a potent antioxidant [47]. Due to high intramuscular creatine concentrations, muscle deposition requires large amounts of this amino acid [48], and creatinine is a chemical waste product of creatine. The higher creatinine levels in the pigs fed the BLEND diet may suggest greater availability of creatine in muscle for use as an antioxidant or for muscle growth. Creatine synthesis requires three amino acids: glycine, arginine, and methionine [49]. The blend probably helped to conserve the use of these amino acids in the synthesis of antioxidants (e.g., glutathione), thus allowing for greater creatine synthesis. However, even though the blend may have allowed for a higher concentration of creatine, it was not able to reduce the effects of HS on protein deposition.

Other biochemical markers also help monitor the effects of HS. At D7, pigs had lower total protein levels compared with the initial conditions, suggesting impaired protein synthesis [50]. The decreased triglyceride levels in our study appear to be related to changes in lipid metabolism under HS. An increase in serum triglyceride levels is associated with an increase in adipose tissue biosynthesis or lipolysis [43]. However, higher levels of insulin during HS limits triglycerides mobilization, thereby constraining lipid mobilization [7]. Interestingly, our results showed lower levels of lactate, which contrasts with the more common HS-induced lactate increase seen in other models [51]. Due to the impaired capability to dissipate heat and the increased body temperature during HS, the animal begins to rely on anaerobic glycolysis in muscle when oxygen becomes limiting, elevating blood lactate as byproduct [7,52]. This discrepancy may be explained by a reduced reliance on anaerobic metabolism by the pigs. Consistent with this, we found lower levels of LDH, suggesting less conversion between lactate and pyruvate [53].

Chronic heat stress (HS) is known to activate the immune system [42], and in the present study, all measured acute-phase proteins (APPs) were affected by HS exposure. Indeed, HS can impair intestinal permeability, thereby triggering immune activation an inflammatory process [6]. The lower circulating glucose levels observed on day 28 may be associated with increased glucose utilization by the immune system [42,54]. Additionally, the lower albumin levels observed may result from a reduction in albumin-dependent fatty acids [32,38]. Although haptoglobin levels typically increase in response to immune activation under HS, our results showed lower levels of haptoglobin. One possible explanation is that environmental challenges do not always elicit strong acute-phase responses, and that lacking of such responses does not necessarily indicate a lack of physiological stress [55,56]. However, our results also showed increased levels of α-1 acid glycoprotein, which support the presence of immune activation, as this protein plays a role in modulating immune and inflammatory responses [57]. Heat stress may also impact immunoglobulin levels. The reduced levels of IgG and IgA at D7 could indicate a weakened humoral immune response. Although cortisol was not assessed in the current study, higher levels of this stress hormone have been associated with lower levels of IgG and potential immunosuppression [58].

In addition to these findings, we expected that blend inclusion might help to alleviate the physiological and metabolic disturbances induced by HS. Chronic HS affects intestinal integrity by increasing its permeability, resulting in a ‘leaky gut’ [42,58]. Several studies have reported that supplementation with natural additives—such as essential oils, yeast metabolites, and herbal blends—can attenuate the negative effects of HS on the gastrointestinal barrier and immune system activation [14,17,59,60]. The beneficial properties of the composition of the blend used in this study (compounds derived from essential oils—carvacrol, eugenol and cinnamaldehyde, yeast metabolites, and capsaicin—has been previously associated with beneficial effects on gut health [13,16], antioxidant and inflammatory status [12], as well as pig performance [17] and thermoregulatory responses [19]. However, in agreement with our results, other studies have shown that supplementation with natural additives did not mitigate the negative consequences of HS [37,61,62,63]. Overall, these findings indicate that the inclusion of the blend had no negative effect on the blood parameters.

## 5. Conclusions

Chronic heat stress consistently induces physiological, metabolic, and immune alterations in pigs. The blend inclusion does not improve pig performance and results in a tendency of lower fat deposition in pigs exposed to thermal challenge. In addition, the blend was associated with higher creatinine concentrations and a trend for increased LDH, which may suggest metabolic adjustments related to muscle energy metabolism. Furthermore, it is important to note that the expected change in performance, body composition, and blood variables was not observed. This lack of effect may be due to several factors such as the dosage of the additive and the duration and intensity of the HS challenge. Given the potential benefits of the natural compounds in the blend, further studies are needed to elucidate their individual and combined mechanisms of action. This could clarify how this feed additive can help pigs better cope with HS and reduce the negative effects of thermal challenge.

## Figures and Tables

**Table 1:** Centesimal and nutritional composition of the experimental diets (as-fed basis).

	Diets			
	Phase 1		Phase 2	
Items	CON	BLEND	CON	BLEND
Ingredients, %				
Corn	73.62	73.62	79.30	79.30
Soybean meal	22.55	22.55	16.54	16.54
Dicalcium phosphate	1.18	1.18	1.06	1.06
Limestone	0.73	0.73	0.71	0.71
Salt	0.25	0.25	0.22	0.22
L-Lysine, 60%	0.32	0.32	0.36	0.36
DL-Methionine, 99%	0.03	0.03	0.02	0.02
L-Threonine, 98.5%	0.01	0.01	0.30	0.30
L-Tryptophan, 98%	-	-	0.01	0.01
L-Valine, 96.5%	-	-	-	-
Choline chloride, 60%	0.06	0.06	0.06	0.06
Premix mineral/vitamin ^1^	0.25	0.25	0.25	0.25
Dextrine	0.50	0.50	0.50	0.50
Soybean oil	0.25	0.25	0.54	0.54
Inert	0.25	-	0.25	-
Feed additive ^2^	-	0.25	-	0.25
Total	100.00	100.00	100.00	100.00
Calculated nutritional composition				
Net Energy, Kcal/kg	2475	2475	2525	2525
Crude protein, %	16.56	16.56	14.27	14.27
SID Lys ^3^, %	0.90	0.90	0.78	0.78
SID Met, %	0.26	0.26	0.23	0.23
SID Met + cys, %	0.51	0.51	0.45	0.45
SID Thp, %	0.54	0.54	0.48	0.48
SID Trp, %	0.17	0.17	0.15	0.15
SID Ile, %	0.61	0.61	0.51	0.51
SID Leu, %	1.35	1.35	1.20	1.20
SID Val, %	0.69	0.69	0.59	0.59
SID Arg, %	0.97	0.97	0.80	0.80
SID Phe, %	1.28	1.28	1.08	1.08
SID His, %	0.40	0.40	0.35	0.35
Ca, %	0.66	0.66	0.60	0.60
Na, %	0.11	0.11	0.10	0.10
Available *p*, %	0.31	0.31	0.28	0.28

^1^ Premix supplied (per kg of diet): vitamin A (7500 UI); vitamin D3 (1562.50 UI); vitamin E (93.75 UI); vitamin K3 (3.13 mg); vitamin B1 (2.19 mg); vitamin B2 (6.25 mg); vitamin B6 (3.75 mg); vitamin B12 (31.25 cmg); vitamin C (0.06 g); folic acid (1.25 mg); pantothenic acid (21.9 mg); biotin (0.22 mg); niacin (43.75 mg); selenium (0.47 mg); copper (125 mg); iron (60 mg); iodine (1.25 mg); manganese (40.63 mg). ^2^ Feed additive composed of compounds derived from essential oils (carvacrol, eugenol, and cinnamaldehyde), pepper extract rich in capsaicin, and yeast metabolites from sugar cane. ^3^ SID: standardized ileal digestible.

**Table 2:** Performance and body composition of growing–finishing gilts fed diets with the addition of an additive composed of a blend of compounds derived from essential oils, pepper extract rich in capsaicin, and yeast metabolites or not under constant heat stress.

	Diets ^1^			
Items	CON	BLEND	SEM ^2^	*p*-Value
Initial conditions				
Initial BW, kg	51.1	51.1	2.48	0.964
Initial body protein, kg	8.35	8.39	0.43	0.780
Initial body lipid, kg	8.03	7.82	0.54	0.509
Phase 1 (0 to 28 days)—Performance and final conditions				
ADFI, kg	1.62	1.53	0.11	0.505
ADG, kg	0.70	0.65	0.04	0.097
G:F	0.44	0.43	0.02	0.890
Protein deposition, g/d	121.2	115.9	7.17	0.397
Protein deposition, % ADG	17.5	17.9	0.52	0.430
Lipid deposition, g/d	120.7	96.2	20.86	0.168
Lipid deposition, % ADG	16.9	14.8	2.39	0.388
Final BW, kg	70.6	69.2	3.71	0.119
Final body protein, kg	11.74	11.64	0.61	0.587
Final body lipid, kg	11.43	10.51	1.07	0.123
Phase 2 (28 to 56 days)—Performance and final conditions				
ADFI, kg	1.92	2.09	0.11	0.260
ADG, kg	0.77	0.78	0.02	0.862
G:F	0.41	0.38	0.02	0.394
Protein deposition, g/d	120.5	128.4	5.44	0.296
Protein deposition, % ADG	16.1	17.0	0.48	0.175
Lipid deposition, g/d	175.6	146.4	16.72	0.174
Lipid deposition, % ADG	23.5	19.7	2.20	0.206
Final BW, kg	91.5	90.2	4.11	0.287
Final body protein, kg	15.12	15.23	0.63	0.686
Final body lipid, kg	16.35	14.61	1.45	0.100
Overall (0 to 56 days)				
ADFI, kg	1.76	1.79	0.11	0.763
ADG, kg	0.72	0.70	0.03	0.338
G:F	0.42	0.40	0.02	0.533
Protein deposition, g/d	120.9	122.1	4.34	0.798
Protein deposition, % ADG	16.8	17.5	0.41	0.125
Lipid deposition, g/d	148.3	121.3	17.18	0.094
Lipid deposition, % ADG	20.4	17.2	1.90	0.141

BW, body weight; ADG, average daily gain; ADFI, average daily feed intake; G:F, gain–feed ratio. ^1^ CON = control diet; BLEND = control diet with inclusion of feed additive composed of a blend of compounds derived from essential oils (carvacrol, eugenol, and cinnamaldehyde), pepper extract rich in capsaicin, and yeast metabolites from sugar cane. ^2^ SEM: standard error of mean.

**Table 3:** Serum and plasma metabolites concentrations of growing–finishing gilts fed diets with the addition of an additive composed of a blend of compounds derived from essential oils, pepper extract rich in capsaicin, and yeast metabolites or not under constant heat stress.

	Diets ^1^				*p*-Value		
Items	CON	BLEND	Mean (Day)	SEM ^2^	Day	Diet	Day × Diet
Total protein, g/dL							
Day 0	6.60	6.61	6.60 ^a^	0.173	<0.001	0.855	0.421
Day 7	3.56	3.34	3.45 ^c^				
Day 28	5.80	6.14	5.97 ^b^				
Mean (Diet)	5.32	5.37					
Urea, mg/dL							
Day 0	20.06	20.17	20.11 ^a^	0.980	<0.001	0.748	0.584
Day 7	13.56	11.78	12.67 ^c^				
Day 28	17.11	17.28	17.19 ^b^				
Mean (Diet)	16.91	16.41					
Creatinine, mg/dL							
Day 0	1.70	1.92	1.81 ^b^	0.053	<0.001	0.032	0.402
Day 7	1.23	1.25	1.24 ^c^				
Day 28	1.95	2.12	2.04 ^a^				
Mean (Diet)	1.63	1.76					
Glucose, mg/dL							
Day 0	73.60	79.67	76.63 ^b^	2.742	<0.001	0.266	0.314
Day 7	82.30	90.17	86.23 ^a^				
Day 28	54.20	50.94	52.57 ^c^				
Mean (Diet)	70.03	73.59					
LDH, U/L							
Day 0	1131.15	1324.78	1227.96 ^a^	46.182	<0.001	0.075	0.218
Day 7	691.40	719.06	705.23 ^c^				
Day 28	994.95	1150.78	1072.86 ^b^				
Mean (Diet)	939.17	1064.87					
Lactate, mg/dL							
Day 0	20.90	24.94	22.92 ^a^	2.089	<0.001	0.758	0.409
Day 7	23.15	24.81	23.98 ^a^				
Day 28	12.70	9.69	11.19 ^b^				
Mean (Diet)	18.92	19.81					
Triglycerides, mg/dL							
Day 0	35.80	37.28	36.54 ^a^	1.724	<0.001	0.879	0.911
Day 7	15.05	14.94	15.00 ^b^				
Day 28	17.85	17.50	17.68 ^b^				
Mean (Diet)	22.90	23.24					

LDH: lactate dehydrogenase. ^1^ CON = control diet; BLEND = control diet with inclusion of feed additive composed of a blend of compounds derived from essential oils (carvacrol, eugenol, and cinnamaldehyde), pepper extract rich in capsaicin, and yeast metabolites from sugar cane. ^2^ SEM: standard error of mean. ^a–c^ Means within a column with different superscripts are affected (*p* < 0.05) by day.

**Table 4:** Serum acute-phase proteins concentrations of growing–finishing gilts fed diets supplemented with a feed additive composed of a blend of compounds derived from essential oils, pepper extract rich in capsaicin, and yeast metabolites or not under constant heat stress.

	Diet ^1^				*p*-Value		
Items	CON	BLEND	Mean (Day)	SEM ^2^	Day	Diet	Day × Diet
Immunoglobulin A, mg/mL							
Day 0	1.28	1.33	1.30 ^a^	0.070	<0.001	0.679	0.987
Day 7	0.78	0.82	0.81 ^b^				
Day 28	1.31	1.36	1.33 ^a^				
Mean (Diet)	1.12	1.16					
Immunoglobulin G, mg/mL							
Day 0	13.61	12.51	13.06 ^b^	0.886	<0.001	0.605	0.595
Day 7	8.99	7.56	8.27 ^c^				
Day 28	15.24	15.38	15.31 ^a^				
Mean (Diet)	12.61	11.82					
Albumin, mg/mL							
Day 0	38.88	39.63	39.25 ^a^	0.812	<0.001	0.597	0.39
Day 7	18.88	17.89	18.38 ^c^				
Day 28	31.23	33.20	32.21 ^b^				
Mean (Diet)	29.66	30.24					
Haptoglobin, mg/dL							
Day 0	0.40	0.40	0.40 ^a^	0.051	<0.001	0.777	0.568
Day 7	0.22	0.12	0.17 ^b^				
Day 28	0.38	0.41	0.40 ^a^				
Mean (Diet)	0.33	0.31					
α-1 Acid glycoprotein, µg/mL							
Day 0	35.70	37.99	36.84 ^b^	3.552	<0.001	0.964	0.889
Day 7	33.39	31.77	32.58 ^b^				
Day 28	49.43	49.50	49.46 ^a^				
Mean (Diet)	39.51	39.75					

^1^ CON = control diet; BLEND = control diet with inclusion of feed additive composed of a blend of compounds derived from essential oils (carvacrol, eugenol, and cinnamaldehyde), pepper extract rich in capsaicin, and yeast metabolites from sugar cane. ^2^ SEM: standard error of mean. ^a–c^ Means within a column with different superscripts are affected (*p* < 0.05) by day.

## Data Availability

The original data presented in this study are openly available in the Institutional Repository of São Paulo State University (UNESP), Faculty of Agricultural and Veterinary Sciences (FCAV), at the following link: https://hdl.handle.net/11449/296441 (accessed on 8 October 2025).

## Abbreviations

The following abbreviations are used in this manuscript:

ADFI	Average Daily-feed Intake
ADG	Average Daily Gain
AIPF	Automatic and Intelligent Precision Feeders
APP	Acute-phase Proteins
BW	Body Weight
CON	Control
DXA	Dual-energy X-ray Absorptiometry
G:F	Gain-to-feed Ratio
HS	Heat Stress
IgA	Immunoglobulin A
IgG	Immunoglobulin B
LHD	Lactate Dehydrogenase
RT	Rectal Temperature
THI	Temperature–humidity Index
TNZ	Thermoneutral Zone
